# The genome sequence of a heleomyzid fly, *Suillia variegata *(Loew, 1862)

**DOI:** 10.12688/wellcomeopenres.19630.1

**Published:** 2023-09-01

**Authors:** Steven Falk, Duncan Sivell, Xavier Richard Badham, University of Oxford and Wytham Woods Genome Acquisition Lab, Natural History Museum Genome Acquisition Lab, Darwin Tree of Life Barcoding collective, Wellcome Sanger Institute Tree of Life programme, Wellcome Sanger Institute Scientific Operations: DNA Pipelines collective, Tree of Life Core Informatics collective, Darwin Tree of Life Consortium

**Affiliations:** 1 Independent researcher, Kenilworth, England, UK; 2 Natural History Museum, London, England, UK; 3 Queen's University Belfast, Belfast, Northern Ireland, UK; 4 University of Aberdeen, Aberdeen, Scotland, UK

**Keywords:** Suillia variegata, a heleomyzid fly, genome sequence, chromosomal, Diptera

## Abstract

We present a genome assembly from an individual male *Suillia variegata* (a heleomyzid fly; Arthropoda; Insecta; Diptera; Heleomyzidae). The genome sequence is 264.0 megabases in span. Most of the assembly is scaffolded into 7 chromosomal pseudomolecules, including the X and Y sex chromosomes. The mitochondrial genome has also been assembled and is 16.17 kilobases in length.

## Species taxonomy

Eukaryota; Metazoa; Eumetazoa; Bilateria; Protostomia; Ecdysozoa; Panarthropoda; Arthropoda; Mandibulata; Pancrustacea; Hexapoda; Insecta; Dicondylia; Pterygota; Neoptera; Endopterygota; Diptera; Brachycera; Muscomorpha; Eremoneura; Cyclorrhapha; Schizophora; Acalyptratae; Sphaeroceroidea; Heleomyzidae; Heleomyzinae; *Suillia*; *Suillia variegata* (Loew, 1862) (NCBI:txid1230148).

## Background

*Suillia variegata* (Loewe, 1862) is a species of fly in the family Heleomyzidae. This species is found all year round throughout the Palearctic region, peaking in density in April/May and then again in July/August. The distribution of *S. variegata* extends across all of Britain and Ireland, with the highest recorded occurrence in England and Wales ( GBIF Secretariat, 2022).

*Suillia variegata* are mycophagous and have been intentionally regularly reared from fungi to great success ( Buxton, 1960; Papp, 1998). However, they have also been successfully reared from decaying flowers, roots and bird’s nests ( Rotheray, 2012). This suggests a mixed feeding strategy around decaying plant matter across a wide range of situations. As a result, *S. variegata* are present in various habitats but are most frequently found in shaded areas near fungi and decaying plant matter. With this in mind, beer traps have been useful in gathering *S. variegata* specimens ( Preisler & Roháček, 2012).

*Suillia variegata* adults have an earthy brown thorax with sparse hairs and a striped abdomen that is pale on the lateral side body. The wings are mostly clear with a small pale area at the apex and a darker area just above. Adults can be distinguished from other Heleomyzidae by the regularly spaced spines on the fore edge of the wings. *Suillia variegata* puparium can be distinguished from other Heleomyzidae with a red-brown ground colour, a wide band of dorsal spicules on abdominal segments 5 to 7 and greater distribution of dorsal spicules posteriorly ( Rotheray, 2012).

The genome of *Suillia variegata* was sequenced as part of the Darwin Tree of Life Project, a collaborative effort to sequence all named eukaryotic species in the Atlantic Archipelago of Britain and Ireland.

## Genome sequence report

**Figure 1.**
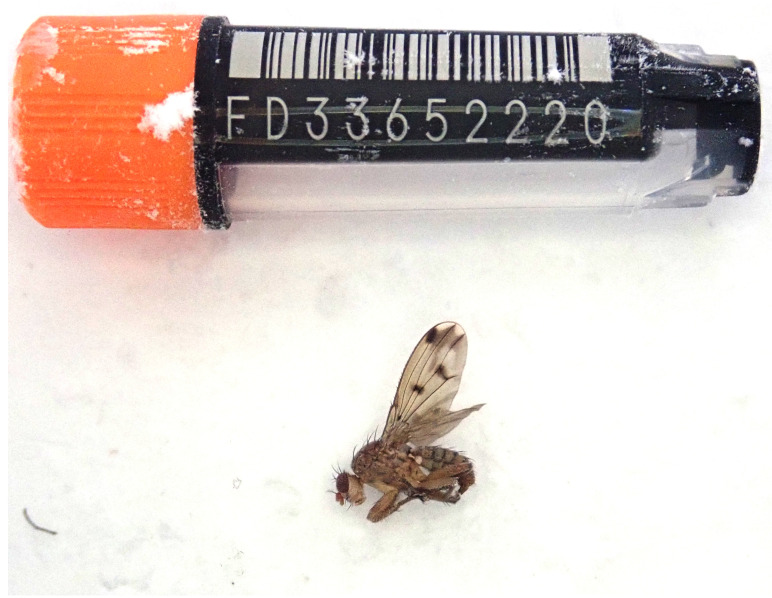
Photograph of the *Suillia variegata* (idSuiVari3) specimen used for genome sequencing.

The genome was sequenced from one male *Suillia variegata* ( Figure 1) collected from Wytham Woods, Oxfordshire, UK (51.77, –1.33). A total of 88-fold coverage in Pacific Biosciences single-molecule HiFi long reads was generated. Primary assembly contigs were scaffolded with chromosome conformation Hi-C data. Manual assembly curation corrected 34 missing joins or mis-joins, reducing the scaffold number by 24.32%.

**Figure 2.**
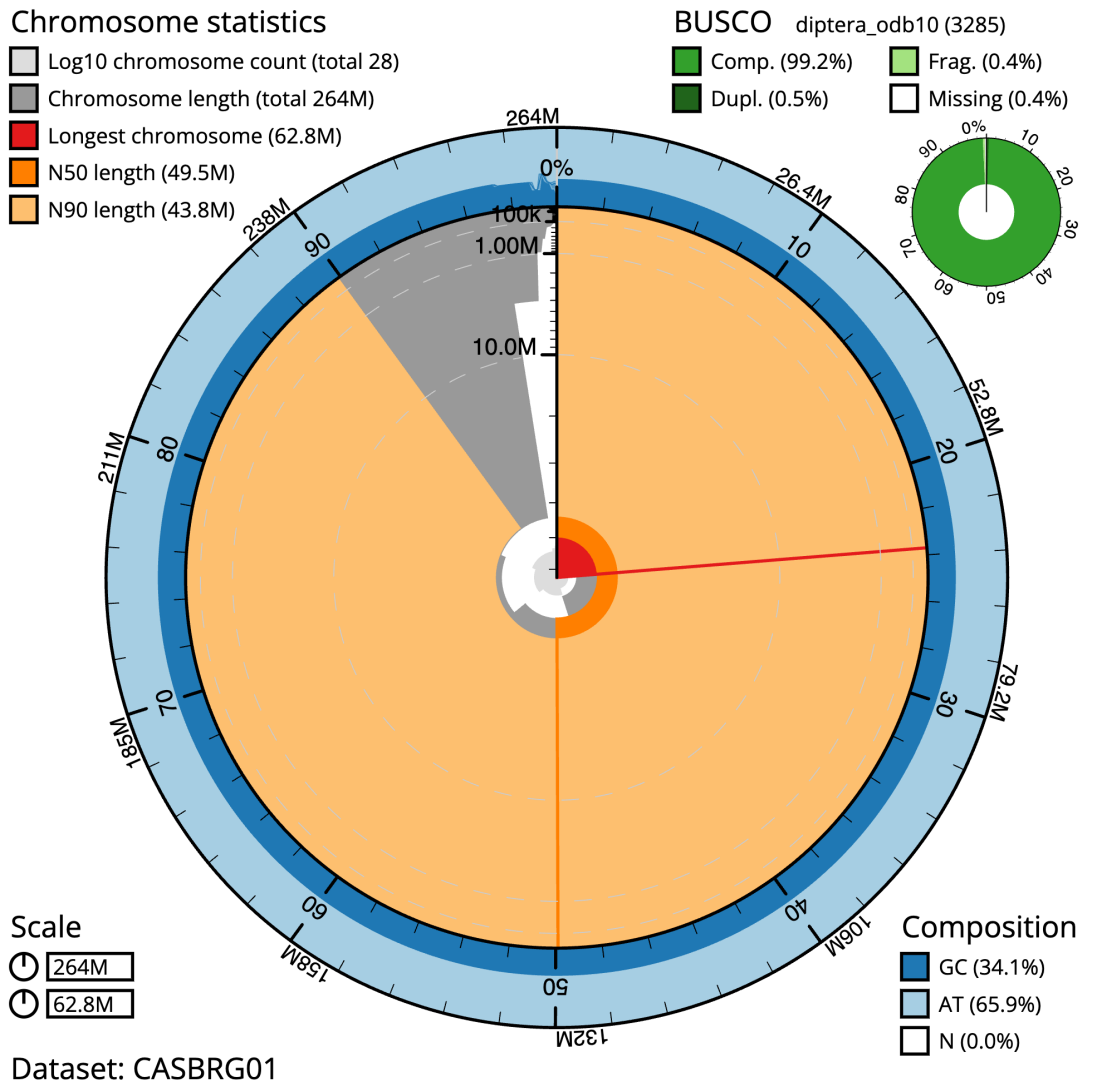
Genome assembly of *Suillia variegata*, idSuiVari3.1: metrics. The BlobToolKit Snailplot shows N50 metrics and BUSCO gene completeness. The main plot is divided into 1,000 size-ordered bins around the circumference with each bin representing 0.1% of the 263,984,139 bp assembly. The distribution of scaffold lengths is shown in dark grey with the plot radius scaled to the longest scaffold present in the assembly (62,750,091 bp, shown in red). Orange and pale-orange arcs show the N50 and N90 scaffold lengths (49,452,834 and 43,836,460 bp), respectively. The pale grey spiral shows the cumulative scaffold count on a log scale with white scale lines showing successive orders of magnitude. The blue and pale-blue area around the outside of the plot shows the distribution of GC, AT and N percentages in the same bins as the inner plot. A summary of complete, fragmented, duplicated and missing BUSCO genes in the diptera_odb10 set is shown in the top right. An interactive version of this figure is available at https://blobtoolkit.genomehubs.org/view/idSuiVari3.1/dataset/CASBRG01/snail.

The final assembly has a total length of 264.0 Mb in 27 sequence scaffolds with a scaffold N50 of 49.5 Mb ( Table 1). Most (99.57%) of the assembly sequence was assigned to seven chromosomal-level scaffolds, representing 5 autosomes and the X and Y sex chromosomes. Chromosome-scale scaffolds confirmed by the Hi-C data are named in order of size ( Figure 2– Figure 5; Table 2). While not fully phased, the assembly deposited is of one haplotype. Contigs corresponding to the second haplotype have also been deposited. The mitochondrial genome was also assembled and can be found as a contig within the multifasta file of the genome submission.

**Table 1.** Genome data for *Suillia variegata*, idSuiVari3.1.

Project accession data		
Assembly identifier	idSuiVari3.1	
Species	*Suillia variegata*	
Specimen	idSuiVari3	
NCBI taxonomy ID	1230148	
BioProject	PRJEB58252	
BioSample ID	SAMEA110451620	
Isolate information	idSuiVari3, male: whole organism (DNA sequencing); idSuiVari2, male: head and thorax (Hi-C scaffolding)	
Assembly metrics *		*Benchmark*
Consensus quality (QV)	60.3	*≥ 50*
*k*-mer completeness	100%	*≥ 95%*
BUSCO **	C:99.2%[S:98.7%,D:0.5%], F:0.4%,M:0.4%,n:3,285	*C ≥ 95%*
Percentage of assembly mapped to chromosomes	99.57%	*≥ 95%*
Sex chromosomes	X and Y	*localised homologous* *pairs*
Organelles	Mitochondrial genome assembled	*complete single alleles*
Raw data accessions		
PacificBiosciences SEQUEL II	ERR10677854	
Hi-C Illumina	ERR10684082	
Genome assembly		
Assembly accession	GCA_949127995.1	
*Accession of alternate* *haplotype*	GCA_949128005.1	
Span (Mb)	264.0	
Number of contigs	179	
Contig N50 length (Mb)	2.8	
Number of scaffolds	27	
Scaffold N50 length (Mb)	49.5	
Longest scaffold (Mb)	62.8	

* Assembly metric benchmarks are adapted from column VGP-2020 of “Table 1: Proposed standards and metrics for defining genome assembly quality” from ( Rhie *et al.*, 2021). ** BUSCO scores based on the diptera_odb10 BUSCO set using v5.3.2. C = complete [S = single copy, D = duplicated], F = fragmented, M = missing, n = number of orthologues in comparison. A full set of BUSCO scores is available at https://blobtoolkit.genomehubs.org/view/idSuiVari3.1/dataset/CASBRG01/busco.

**Figure 3.**
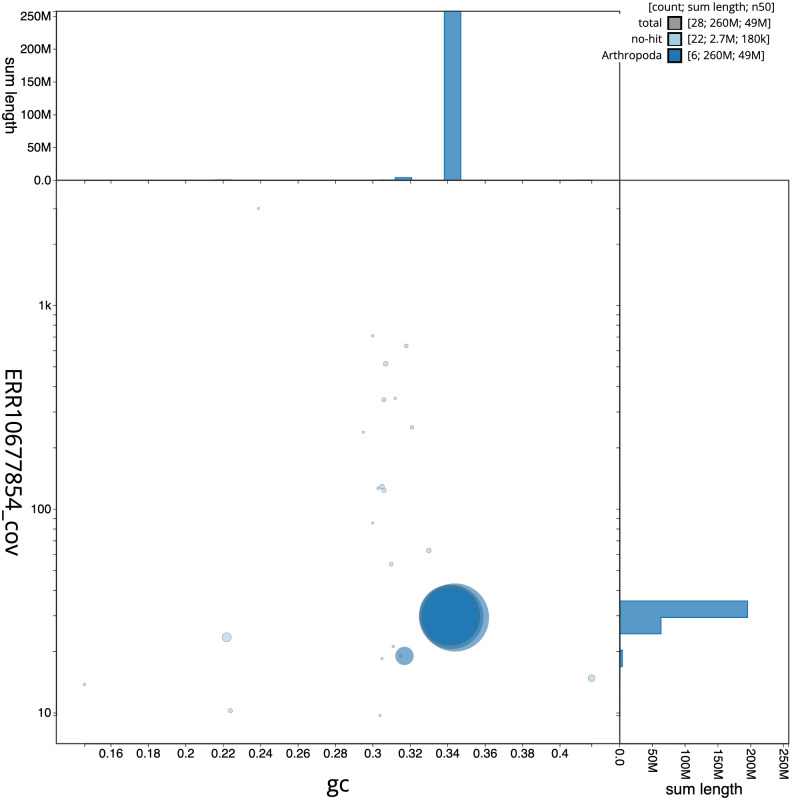
Genome assembly of *Suillia variegata*, idSuiVari3.1: BlobToolKit GC-coverage plot. Scaffolds are coloured by phylum. Circles are sized in proportion to scaffold length. Histograms show the distribution of scaffold length sum along each axis. An interactive version of this figure is available at https://blobtoolkit.genomehubs.org/view/idSuiVari3.1/dataset/CASBRG01/blob.

**Figure 4.**
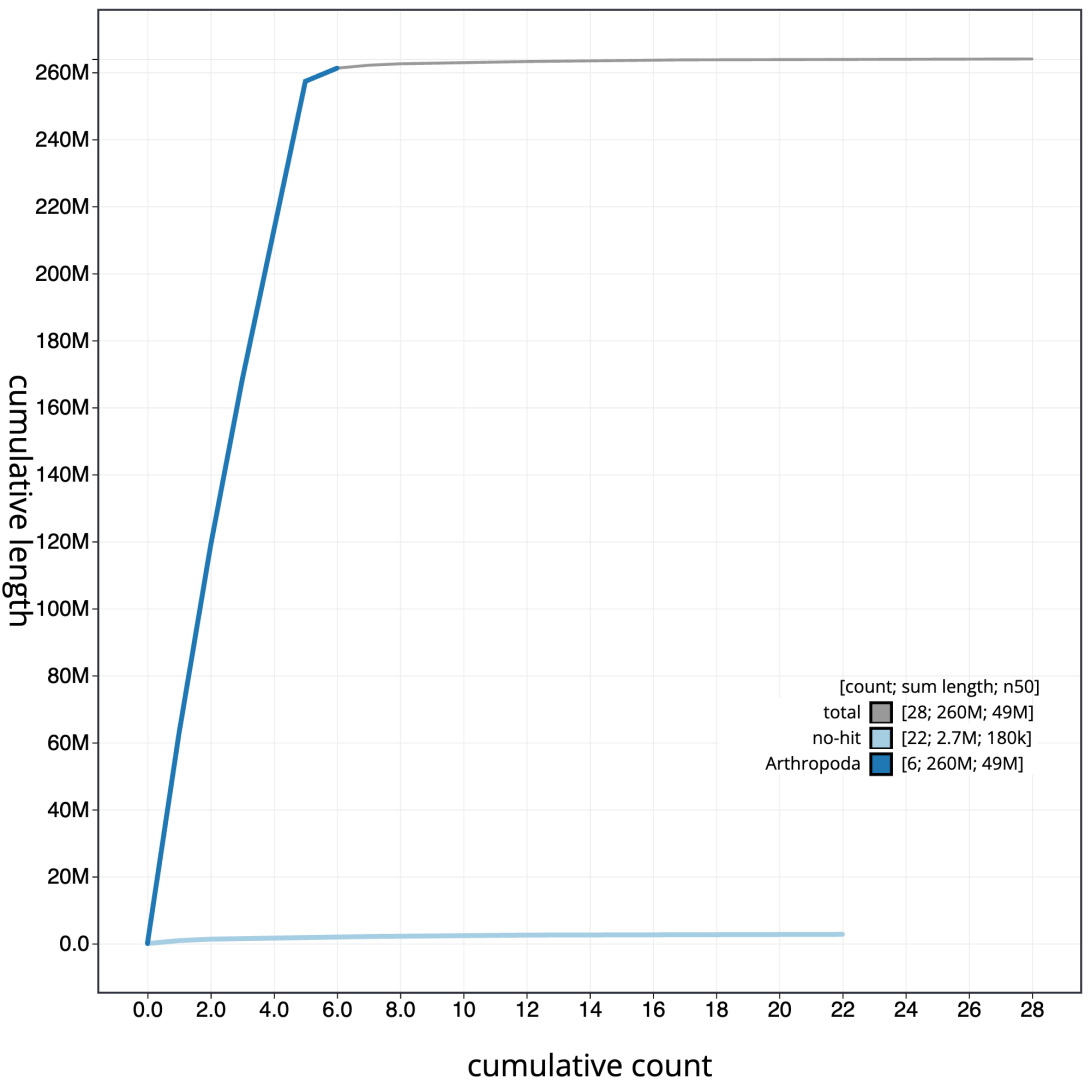
Genome assembly of *Suillia variegata*, idSuiVari3.1: BlobToolKit cumulative sequence plot. The grey line shows cumulative length for all scaffolds. Coloured lines show cumulative lengths of scaffolds assigned to each phylum using the buscogenes taxrule. An interactive version of this figure is available at https://blobtoolkit.genomehubs.org/view/idSuiVari3.1/dataset/CASBRG01/cumulative.

**Figure 5.**
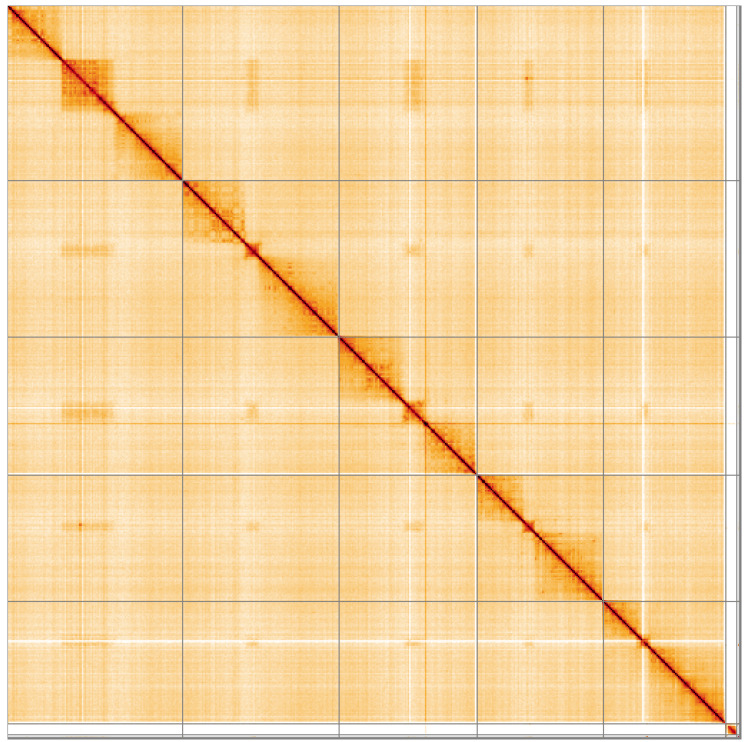
Genome assembly of *Suillia variegata*, idSuiVari3.1: Hi-C contact map of the idSuiVari3.1 assembly, visualised using HiGlass. Chromosomes are shown in order of size from left to right and top to bottom. An interactive version of this figure may be viewed at https://genome-note-higlass.tol.sanger.ac.uk/l/?d=ZA72LrlPSAOQgCpfERxzXw.

**Table 2.** Chromosomal pseudomolecules in the genome assembly of *Suillia variegata*, idSuiVari3.

INSDC accession	Chromosome	Length (Mb)	GC%
OX421842.1	1	62.75	34.5
OX421843.1	2	56.03	34.0
OX421844.1	3	49.45	34.0
OX421845.1	4	45.24	34.0
OX421846.1	5	43.84	34.0
OX421847.1	X	3.92	31.5
OX421848.1	Y	0.43	41.5
OX421849.1	MT	0.02	24.0

The estimated Quality Value (QV) of the final assembly is 60.3 with *k*-mer completeness of 100%%, and the assembly has a BUSCO v5.3.2 completeness of 99.2% (single = 98.7%, duplicated = 0.5%), using the diptera_odb10 reference set ( *n* = 3,285).

Metadata for specimens, spectral estimates, sequencing runs, contaminants and pre-curation assembly statistics can be found at https://links.tol.sanger.ac.uk/species/1230148.

## Methods

### Sample acquisition and nucleic acid extraction

A male *Suillia variegata* (specimen ID Ox002179, ToLID idSuiVari3) was collected from Wytham Woods, Oxfordshire (biological vice-county Berkshire), UK (latitude 51.77, longitude –1.33) on 2022-05-19. The specimen was collected and identified by Steven Falk (independent researcher) and preserved on dry ice.

The specimen used for Hi-C scaffolding was a male *S. variegata* (specimen ID NHMUK014449032, ToLID idSuiVari2), collected and identified by Duncan Sivell (Natural History Museum) from the Natural History Museum Wildlife Garden on 2021-04-21. This specimen was dry frozen at –80°C.

The idSuiVari3 sample was prepared for DNA extraction at the Tree of Life laboratory, Wellcome Sanger Institute (WSI). The sample was weighed and dissected on dry ice with tissue set aside for Hi-C sequencing. Tissue from the whole organism was disrupted using a Nippi Powermasher fitted with a BioMasher pestle *.* DNA was extracted at the WSI Scientific Operations core using the Qiagen MagAttract HMW DNA kit, according to the manufacturer’s instructions.

### Sequencing

Pacific Biosciences HiFi circular consensus DNA sequencing libraries were constructed according to the manufacturers’ instructions. DNA sequencing was performed by the Scientific Operations core at the WSI on a Pacific Biosciences SEQUEL II (HiFi) instrument. Hi-C data were also generated from head and thorax tissue of idSuiVari2 using the Arima2 kit and sequenced on the Illumina NovaSeq 6000 instrument.

### Genome assembly, curation and evaluation

Assembly was carried out with Hifiasm ( Cheng *et al.*, 2021) and haplotypic duplication was identified and removed with purge_dups ( Guan *et al.*, 2020). The assembly was then scaffolded with Hi-C data ( Rao *et al.*, 2014) using YaHS ( Zhou *et al.*, 2023). The assembly was checked for contamination and corrected using the gEVAL system ( Chow *et al.*, 2016) as described previously ( Howe *et al.*, 2021). Manual curation was performed using gEVAL, HiGlass ( Kerpedjiev *et al.*, 2018) and Pretext ( Harry, 2022). The mitochondrial genome was assembled using MitoHiFi ( Uliano-Silva *et al.*, 2022), which runs MitoFinder ( Allio *et al.*, 2020) or MITOS ( Bernt *et al.*, 2013) and uses these annotations to select the final mitochondrial contig and to ensure the general quality of the sequence.

A Hi-C map for the final assembly was produced using bwa-mem2 ( Vasimuddin *et al.*, 2019) in the Cooler file format ( Abdennur & Mirny, 2020). To assess the assembly metrics, the *k*-mer completeness and QV consensus quality values were calculated in Merqury ( Rhie *et al.*, 2020). This work was done using Nextflow ( Di Tommaso *et al.*, 2017) DSL2 pipelines “sanger-tol/readmapping” ( Surana *et al.*, 2023a) and “sanger-tol/genomenote” ( Surana *et al.*, 2023b). The genome was analysed within the BlobToolKit environment ( Challis *et al.*, 2020) and BUSCO scores ( Manni *et al.*, 2021; Simão *et al.*, 2015) were calculated.

Table 3 contains a list of relevant software tool versions and sources.

**Table 3.** Software tools: versions and sources.

Software tool	Version	Source
BlobToolKit	4.1.7	https://github.com/blobtoolkit/ blobtoolkit
BUSCO	5.3.2	https://gitlab.com/ezlab/busco
gEVAL	N/A	https://geval.org.uk/
Hifiasm	0.16.1-r375	https://github.com/chhylp123/ hifiasm
HiGlass	1.11.6	https://github.com/higlass/higlass
Merqury	MerquryFK	https://github.com/thegenemyers/ MERQURY.FK
MitoHiFi	2	https://github.com/marcelauliano/ MitoHiFi
PretextView	0.2	https://github.com/wtsi-hpag/ PretextView
purge_dups	1.2.3	https://github.com/dfguan/purge_ dups
sanger-tol/ genomenote	v1.0	https://github.com/sanger-tol/ genomenote
sanger-tol/ readmapping	1.1.0	https://github.com/sanger-tol/ readmapping/tree/1.1.0
YaHS	1.2a	https://github.com/c-zhou/yahs

### Wellcome Sanger Institute – Legal and Governance

The materials that have contributed to this genome note have been supplied by a Darwin Tree of Life Partner. The submission of materials by a Darwin Tree of Life Partner is subject to the **‘Darwin Tree of Life Project Sampling Code of Practice’**, which can be found in full on the Darwin Tree of Life website here. By agreeing with and signing up to the Sampling Code of Practice, the Darwin Tree of Life Partner agrees they will meet the legal and ethical requirements and standards set out within this document in respect of all samples acquired for, and supplied to, the Darwin Tree of Life Project.

Further, the Wellcome Sanger Institute employs a process whereby due diligence is carried out proportionate to the nature of the materials themselves, and the circumstances under which they have been/are to be collected and provided for use. The purpose of this is to address and mitigate any potential legal and/or ethical implications of receipt and use of the materials as part of the research project, and to ensure that in doing so we align with best practice wherever possible. The overarching areas of consideration are: 

•   Ethical review of provenance and sourcing of the material

•   Legality of collection, transfer and use (national and international) 

Each transfer of samples is further undertaken according to a Research Collaboration Agreement or Material Transfer Agreement entered into by the Darwin Tree of Life Partner, Genome Research Limited (operating as the Wellcome Sanger Institute), and in some circumstances other Darwin Tree of Life collaborators.

## Data Availability

European Nucleotide Archive: *Suillia variegata*. Accession number PRJEB58252; https://identifiers.org/ena.embl/PRJEB58252. ( Wellcome Sanger Institute, 2022) The genome sequence is released openly for reuse. The *Suillia variegata* genome sequencing initiative is part of the Darwin Tree of Life (DToL) project. All raw sequence data and the assembly have been deposited in INSDC databases. The genome will be annotated using available RNA-Seq data and presented through the Ensembl pipeline at the European Bioinformatics Institute. Raw data and assembly accession identifiers are reported in Table 1.

## Author information

Members of the University of Oxford and Wytham Woods Genome Acquisition Lab are listed here: https://doi.org/10.5281/zenodo.4789928.

Members of the Natural History Museum Genome Acquisition Lab are listed here:

https://doi.org/10.5281/zenodo.4790042.

Members of the Darwin Tree of Life Barcoding collective are listed here: https://doi.org/10.5281/zenodo.4893703.

Members of the Wellcome Sanger Institute Tree of Life programme are listed here: https://doi.org/10.5281/zenodo.4783585.

Members of Wellcome Sanger Institute Scientific Operations: DNA Pipelines collective are listed here: https://doi.org/10.5281/zenodo.4790455.

Members of the Tree of Life Core Informatics collective are listed here: https://doi.org/10.5281/zenodo.5013541.

Members of the Darwin Tree of Life Consortium are listed here: https://doi.org/10.5281/zenodo.4783558.
