# Supplementary material for: The genome sequence of a heleomyzid fly, Suillia variegata (Loew, 1862)
Source: Wellcome Open Res. 2023 Sep 1;8:382. [Version 1] doi: 10.12688/wellcomeopenres.19630.1 (PMC11319907; doi:10.12688/wellcomeopenres.19630.1)
Supplement: Peer review history

# Reviewer report: Reviewer response for version 1

**DOI:** 10.21956/wellcomeopenres.21746.r89663

**Published:** 2024-08-12

**Authors:** Rodolpho S.T Menezes

**Affiliations:** 1 Department of Biological Sciences, State University of Santa Cruz, Ilhéus, Brazil

The data note by Falk et al. presents a genome assembly from male of the heleomyzid fly, *Suillia variegata* (Loew, 1862). The final assembly is 264 Mb. This genome information will be interesting for comparative genomics investigation and also for evolution and conservation studies. The methodology is thorough and explained and the figures are well constructed and the data note is well written.

 I have no notes to add.

Are sufficient details of methods and materials provided to allow replication by others?

Yes

Is the rationale for creating the dataset(s) clearly described?

Yes

Are the datasets clearly presented in a useable and accessible format?

Yes

Are the protocols appropriate and is the work technically sound?

Yes

Reviewer Expertise:

Molecular systematics, Phylogenomics, Cytogenetics, and Phylogeography.

I confirm that I have read this submission and believe that I have an appropriate level of expertise to confirm that it is of an acceptable scientific standard.

# Reviewer report: Reviewer response for version 1

**DOI:** 10.21956/wellcomeopenres.21746.r89661

**Published:** 2024-08-08

**Authors:** Darren J Obbard

**Affiliations:** 1 University of Edinburgh, Edinburgh EH, UK

This data note reports the sequencing and assembly of the genome of *Suillia variegata* as part of the “Darwin Tree of Life” programme. In common with other data notes from this research effort, the reporting is standardised and quite brief. As such, I have very few comments to make.

 The approach is state-of-the-art, the raw data appear to be of a suitably high quality, and the assembly methods are appropriate. The public availability of raw data and genome assembly are appropriate. The resulting genome is likely to be of very high quality, and I have no doubt that it will be of great value to any researchers working on the comparative or evolutionary genomics of insects.

 I really have almost no suggestions for improvement: I usually suggest cross-references to the research literature – but I really cannot find any that were missed.

1. I suggest including some better photos of the species to provide context – ideally male and female in life.  'I’m sure the first author must have some available.'
2. It would be good to say something about the global distribution, to put the UK sample in perspective.
3. The percentage sign is duplicated in “…of the final assembly is 60.3 with k-mer completeness of 100%% …”

Are sufficient details of methods and materials provided to allow replication by others?

Yes

Is the rationale for creating the dataset(s) clearly described?

Yes

Are the datasets clearly presented in a useable and accessible format?

Yes

Are the protocols appropriate and is the work technically sound?

Yes

Reviewer Expertise:

Evolutionary genetics of invertebrates, especially Diptera, and their pathogens

I confirm that I have read this submission and believe that I have an appropriate level of expertise to confirm that it is of an acceptable scientific standard.
